# Editorial: Sociology of emotion and affect in the age of mis-, dis-, and mal-information

**DOI:** 10.3389/fsoc.2025.1563408

**Published:** 2025-03-05

**Authors:** Alberto Bellocchi, Timothy Graham, Stina Bergman Blix, Darren Linvill

**Affiliations:** ^1^Learning Science and Innovation Laboratory, Queensland University of Technology, Brisbane, QLD, Australia; ^2^Digital Media Research Centre, Queensland University of Technology, Brisbane, QLD, Australia; ^3^Department of Sociology, Uppsala University, Uppsala, Sweden; ^4^Department of Communication, Clemson University, Clemson, SC, United States

**Keywords:** sociology of emotion, misinformation, disinformation, online, affective

## 1 Introduction

In recent public discourse, particularly with elections in powerful countries, the term *disinformation* has entered our lexicon. Although the intentional spread of incorrect information is likely to be older than the printing press, it is widely acknowledged that the internet, social media, and most recently, artificial intelligence tools, have increased the capacity for people to spread disinformation rapidly and to any individual or group with access to the internet (cf. Vosoughi et al., 2018). For the purposes of this editorial, we follow Wardle and Derakhshan (2017) in defining disinformation as the intentional spread of incorrect information, misinformation as the unintentional spread, and malinformation as the intentional spread of incorrect information designed to cause harm. For simplicity, we adopt disinformation as the umbrella term.

We understand emotions to be multi-componential aspects of human experience, both individual and social, that involve biological and cultural processes involving physiological states of arousal (Turner, 2007). In a wide range of fields of inquiry, emotions are understood to have components including bodily gestures (e.g., slumped posture) and expressions (e.g., facial expressions such as a smile), physiological states (e.g., pupil dilation, increased heart rate), linguistic labels (anger, fear, joy, disgust), and situational cues (e.g., a person's reaction to what another says or does). Although the field of sociology of emotion is relatively young compared to other traditions, scholarship has burgeoned in the last 10 years (cf. Patulny et al., 2019). It is timely to bring together scholars from a range of disciplines to consider the intersection of sociology of emotion with disinformation.

A study of false news stories shared on X (formerly Twitter) between 2006–2017 is perhaps emblematic of the way in which the internet and social media facilitates the spread of disinformation (Vosoughi et al., 2018). Focusing on rumor cascades, which commence when a user states a claim that can include multi-media, social media facilitates others to propagate this claim by resharing it. Vosoughi et al. (2018) identified ~126,000 rumor cascades spread by 3 million people over 4.5 million times. Using fact-checking organizations, the claims have been evaluated to determine their veracity. The researchers conclude that disinformation, in the form of false news, spreads faster, farther, and travels deeper into social networks when compared to true information. In addition to the range and speed of disinformation, the study also establishes connections to emotions. The authors find that replies to false stories inspire surprise, fear, and disgust, whereas true stories are met with inspired anticipation, joy, and trust. In this way, research evidence establishing the interplay between emotion and disinformation disseminated via social networks is beginning to emerge.

Other research has associated rating features, such as upvotes/downvotes, of social media with emotions (Davis and Graham, 2021). Analyzing a social media news site that utilizes binary ratings of posts (upvote/downvote), and how participants vote on posts, Davis and Graham (2021) find that upvotes precede positive emotions in subsequent posts, and downvotes precede negative emotions. In this way, vote scores are predictive of emotions expressed in posts, and, more importantly, negative emotions foster greater active engagement with posts. Pertinent to disinformation, the study reveals that downvoted content receives higher levels of engagement than upvoted content. This finding suggests, by inference, that more interaction occurs over negative emotions than positive emotions, however other research produces mixed results (cf. Ferrara and Yang, 2015; Gruzd, 2013; Stieglitz and Dang-Xuan, 2013). We can expect that disinformation attracts considerable emotional purchase, and that there is a likelihood for negative information and emotion to engender considerable active engagement with content based on this prior work.

Vosoughi et al. and Davis and Graham's studies point to the ways in which emotions are relevant to human online interactions. As the studies in this Research Topic will show, social media is but one of the means via which emotion and disinformation come together in social life.

## 2 Contributions to the Research Topic

In responding to our call for papers on the *Sociology of Emotion in the Age of Mis-, Dis-, and Mal-information*, we received contributions addressing robotic misinformation in a dementia care context (Persson et al.), misinformation in the humanitarian response to Muslim refugees (Khorana and Thapliyal), and the manipulative capacity of emotional Artificial Intelligence (Bakir et al.). The diversity of topics represented speaks to the lack of boundaries for disinformation; there is no place, space, topic, or person who may remain untouched.

### 2.1 Emotional dimensions of information disorder in media ecologies of refugees and asylum seekers

Khorana and Thapliyal make a compelling contribution to the topic through their multi-country study of “information disorder” in shaping perceptions and treatment of Muslim refugees and asylum seekers in India and Australia. The paper introduces a novel concept—“calculated care”—that captures how compassion is selectively applied based on religious and cultural biases. In doing so they highlight how approaches that seem humanitarian can be deeply discriminatory. The authors look at the role of disinformation—often spread by the state and propagated in hybrid media systems—that mobilizes and exploits emotions such as fear, resentment, and distrust, fostering an affective environment that legitimizes and affords mistreatment of Muslim refugees. By forging this link between emotion and disinformation, Khorana and Thapliyal contribute a much-needed dimension to the sociology of emotion and disinformation studies, shining a light on how affect can be weaponised to entrench power and maintain sociopolitical boundaries.

Through a layered analysis of “information disorder,” the study examines how officials, mainstream media, and social media platforms jointly function to produce disinformation. The systemic structure of this hybrid media system brings to attention the coordinated and deliberate aspects of misleading the public, where state and non-state actors alike perpetuate discourses that construct Muslim refugees and asylum seekers as unworthy of care. By focusing on the unique vulnerabilities and experiences faced by Muslim refugees within a systemic media environment of Islamophobia, Khorana and Thapliyal offer new knowledge and perspectives on how humanitarianism is itself selectively enacted. This challenges the idea of humanitarian care as a uniformly applied practice in the context of global displacement. Likewise, by positioning emotion as central to the spread and discursive entrenchment of disinformation, the authors make available a new space for scholars to explore this critical area of research in the 21st century.

### 2.2 Emotional AI and the potential for manipulation

Bakir et al. work to understand whether and why people have concerns about potential manipulation resulting from AI emotional profiling. In other words, do people care and, if so, why do they care that AI might analyze their emotional state and use the information learned to promote engagement and shape individual behavior? To explore these questions their work engaged in a two-part research study.

Stage one employed focus groups to uncover the kinds of concerns people had about emotion profiling. Here they found participants frequently expressed concern about how individual weaknesses and vulnerabilities might be exploited by AI in such a way that might damage individuals' capacity for rational thought and action. Stage two of the research adopted a demographically representative survey of the United Kingdom. Most participants expressed genuine concerns about the potential of being manipulated by AI through social media and emotoys.

In addressing the risks of AI addressed in their work, the authors acknowledge a complex and technically difficult task. They suggest, however, that their findings indicate the public may have a desire for strong social protections related to AI's use in emotion profiling. It is, they argue, up to a combination of public regulation and the work of socially minded developers to seek forms of engagement that do not rely on automated emotion profiling.

### 2.3 Social robots and misinformation in health care

Persson et al. turn the problem with misinformation on its head in their analysis of the use of pet robots in dementia care. In some situations, the truth about a pet robot's status as fake seems to be less important. Ethical guidelines require that robots used in care practice are introduced as robots, but their empirical study on five dementia care homes in Sweden showed that care workers instead used emotional cues from the residents to allow for the residents themselves to interpret the status of the pet robots. Some interpreted the pet as real, some as fake and some had a more ambiguous approach. The care workers' alertness to emotional cues put the relation building between the pet and the resident in focus. Whether the resident interpreted the pet robot as real or fake did not necessarily influence their interaction with it. Sometimes this strategy backfired and the residents got angry from feeling manipulated to play with a fake animal, but several residents engaged in active interpretation to remain in an ambiguous relation to the pet, seemlingly similar to children's play with dolls or stuffed animals. The realness of the doll, or in this case the pet robot, was not important or, at least, set aside for the time being, so that they could play or cuddle with it “as if” it was real.

The paper highlights the importance of emotions for evaluating robotic misinformation in a care context and the active involvement in sense making by people with dementia. Emotions are crucial when residents negotiate their relation to the pet robot, and provide important cues for the care workers to encourage residents' own interpretations. In this context, misinformation, or whether the robot is real or fake, can be less important than the patients' ability to create meaning on their own terms.

## 3 Conclusion

*Sociology of emotion and affect in the age of mis-, dis-, and mal-information* is a topic for our time given the nature of social and online landscape that we live in. With recent widespread adoption of machine learning technology in commonly used tools such as internet search engines, mobile devices, and home devices, the affordances and challenges society faces with respect to false information will be with us indefinitely. Perhaps this Research Topic is ahead of its time, signaling the challenge that research scholarship faces in the wake of such susbtantive and influential change. As the contributions to the topic reveal, emotion and disinformation are inextricably linked to our uses of technology in contexts as diverse as reporting about refugees (Khorana and Thapliyal), emotional profiling by Artificial Intelligence (Bakir et al.), and robots in aged-care (Persson et al.). The articles in this Research Topic demonstrate how disinformation can be understood across such diverse contexts from the vatange point of the sociology of emotions and affect, a relevatively recent field which continues to gain rapid traction. Across the articles in this Research Topic, we see a shift in the associations between emotion, technology, and dinsinformation. Khorana and Thapliyal reveal how the spread of disinformation via media systems shapes affective environments related to refugees. Bakir et al.'s work reveals a different association as AI technology is used for emotional profiling, creating a need for society to respond by regulating AI use with respect to the emotions. In Persson et al.'s study we see how care-workers interpret emotional responses of dementia care patients when they interact with pet robots, which are considered forms of misinformation. Each study contains far more nuance than what this editorial can capture, and we invite readers to consider carefully in reading each article how emotion, disinformation, and technology interplay. This Research Topic should spark healthy new directions for research and more specifically the Sociology of Emotion and Affect, which holds promise as we advance further into a future in which society, technology, emotion, and information continue a relationship as old as our species.

## References

[B1] Davis J. L. Graham T. (2021). Emotional consequences and attention rewards: the social effects of rating on Reddit. Inf. Commun. Soc. 25, 649–666. 10.1080/1369118X.2021.1874476

[B2] Ferrara E. Yang Z. (2015). Quantifying the effect of sentiment on information diffusion in social media. Peer J. Comput. Sci. 1:e26. 10.7717/peerj-cs.2633529153

[B3] Gruzd A. (2013). Emotions in the twitterverse and implications for user interface design. AIS Trans. Hum.-Comput. Interact. 5, 42–56. 10.17705/1thci.00053

[B4] Patulny R. Olson R. Khorana S. McKenzie J. Bellocchi A. (2019). “Introduction,” in Emotions in Late Modernity, eds. R. Patulny, A. Bellocchi, R. Olson, S. Khorana, J. McKenzie, and M. Peterie (London: Routledge), 1.

[B5] Stieglitz S. Dang-Xuan L. (2013). Emotions and information diffusion in social media – sentiment of microblogs and sharing behavior. J. Manag. Inf. Syst. 29, 217–248. 10.2753/MIS0742-1222290408

[B6] Turner J. H. (2007). Human Emotions: A Sociological Theory. London: Routledge.

[B7] Vosoughi S. Roy D. Aral S. (2018). The spread of true and false news online. Science 359, 1146–1151. 10.1126/science.aap9559 29590045

[B8] Wardle C. Derakhshan H. (2017). Information Disorder: Toward A Transdisciplinary Framework for Research and Policymaking. Strasbourg: Council of Europe.

